# Supplementary material for: The non-coding RNA landscape of human hematopoiesis and leukemia
Source: Nat Commun. 2017 Aug 9;8:218. doi: 10.1038/s41467-017-00212-4 (PMC5550424; doi:10.1038/s41467-017-00212-4)
Supplement: Supplementary file 1 — Supplementary Information [file 41467_2017_212_MOESM1_ESM.pdf]

Title of file for HTML: Supplementary Information
Description: Supplementary Figures, Supplementary Table and Supplementary References

Title of file for HTML: Supplementary Data 1
Description: Top lineage specific ncRNAs from the Arraystar Arraystar lncRNA V2.0 platform

Title of file for HTML: Supplementary Data 2
Description: Top lineage specific ncRNAs from the NCode™ ncRNA platform

Title of file for HTML: Supplementary Data 3
Description: Custom hematopoietic gene sets used for GSEA

Title of file for HTML: Supplementary Data 4
Description: HOTAIRM1 guilt-by-association gene sets

Title of file for HTML: Supplementary Data 5
Description: SOM-modules of ncRNAs from the RNA-seq platform

Title of file for HTML: Supplementary Data 6
Description: Top lineage specific miRNAs from the NCode™ miRNA platform

Title of file for HTML: Supplementary Data 7
Description: ncRNA signatures used as input for clustering the TCGA dataset

Title of file for HTML: Supplementary Data 8
Description: shRNAs and sgRNAs

Title of file for HTML: Peer Review File
Description:

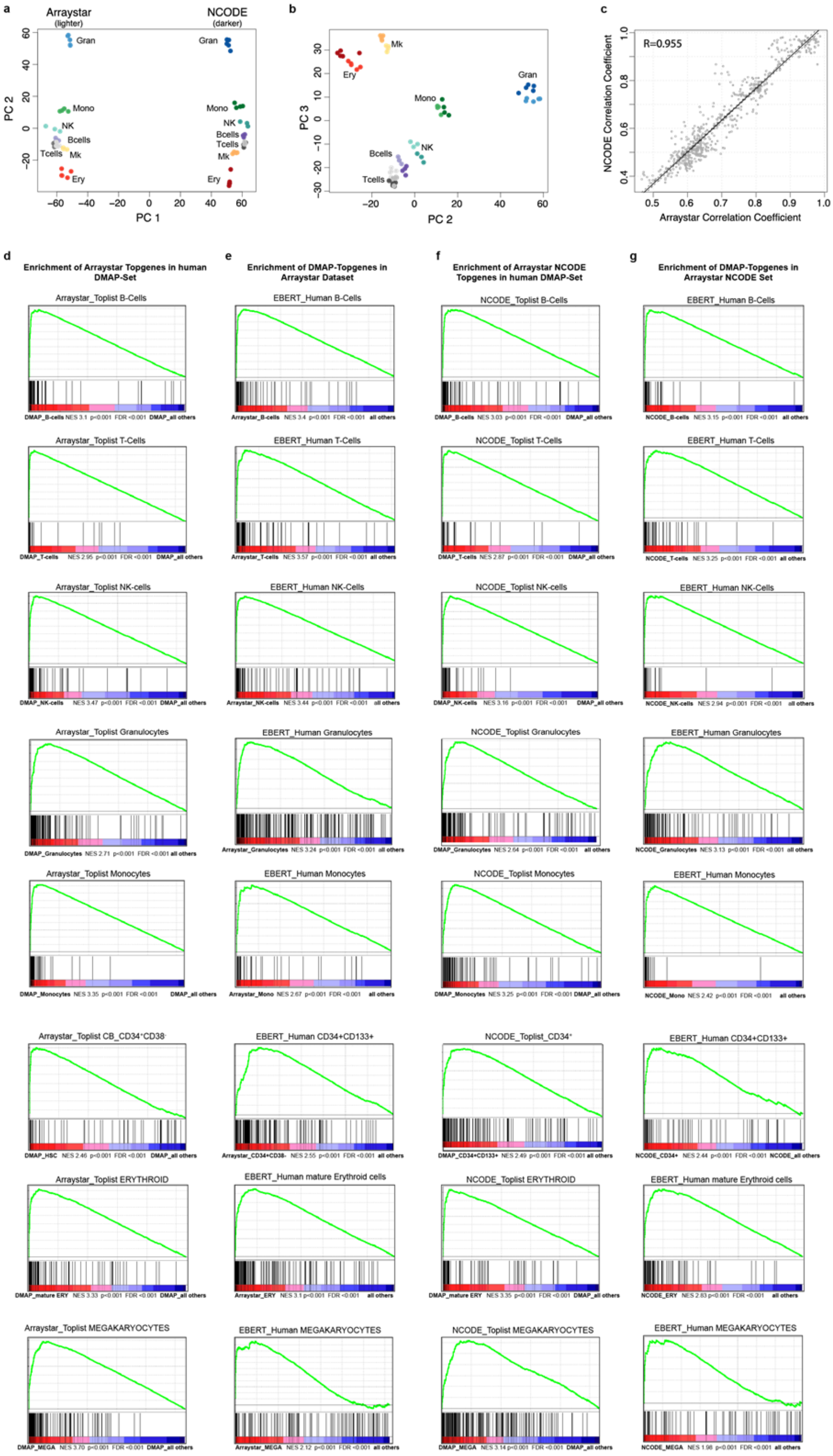

a
Arraystar (lighter)
NCODE (darker)
Gran
Mono
NK
Bcells
Tcells
Mk
Ery
PC 1
PC 2
b
PC 2
PC 3
c
R=0.955
Arraystar Correlation Coefficient
NCODE Correlation Coefficient
d
Enrichment of Arraystar Topgenes in human DMAP-Set
e
Enrichment of DMAP-Topgenes in Arraystar Dataset
f
Enrichment of Arraystar NCODE Topgenes in human DMAP-Set
g
Enrichment of DMAP-Topgenes in Arraystar NCODE Set
Arraystar_Toplist B-Cells
EBERT_Human B-Cells
NCODE_Toplist B-Cells
Arraystar_Toplist T-Cells
EBERT_Human T-Cells
NCODE_Toplist T-Cells
Arraystar_Toplist NK-cells
EBERT_Human NK-Cells
NCODE_Toplist NK-cells
Arraystar_Toplist Granulocytes
EBERT_Human Granulocytes
NCODE_Toplist Granulocytes
Arraystar_Toplist Monocytes
EBERT_Human Monocytes
NCODE_Toplist Monocytes
EBERT_Human CD34+CD133+
NCODE_Toplist_CD34+
Arraystar_Toplist ERYTHROID
EBERT_Human mature Erythroid cells
NCODE_Toplist ERYTHROID
Arraystar_Toplist MEGAKARYOCYTES
EBERT_Human MEGAKARYOCYTES
NCODE_Toplist MEGAKARYOCYTES

Supplementary Fig. 1. Platform and data set cross validation via PCA and GSEA on top lineage-specific genes.
(**a**) PCA on 15,219 GENCODE-annotated transcripts represented on the Arraystar ncRNA Array V2.0 and NCode™-ncRNA arrays: PC1 (62% of the total variance) separates the samples based on the technical platform. (**b**) PC2 and PC3 clustered samples from different platforms according to cell type. (**c**) Correlation of correlations between all possible pairwise sample combinations on each platform as parameter of global concordance.[1] (**d**) The top lineage-specific coding genes from the Arraystar ncRNA Array V2.0 platform tested for enrichment in the respective populations of the human DMAP dataset.[2] (**e**) The top lineage-specific coding genes from the human DMAP dataset tested for enrichment in the respective populations of the Arraystar ncRNA Array V2.0 dataset. (**f**) The top lineage-specific coding genes from the NCode™-ncRNA platform tested for enrichment in the respective populations of the human DMAP dataset. (**g**) The top lineage-specific coding genes from the human DMAP dataset tested for enrichment in the respective populations of the NCode™-ncRNA dataset. All gene sets used for the analysis are in **Supplementary Data 3**.

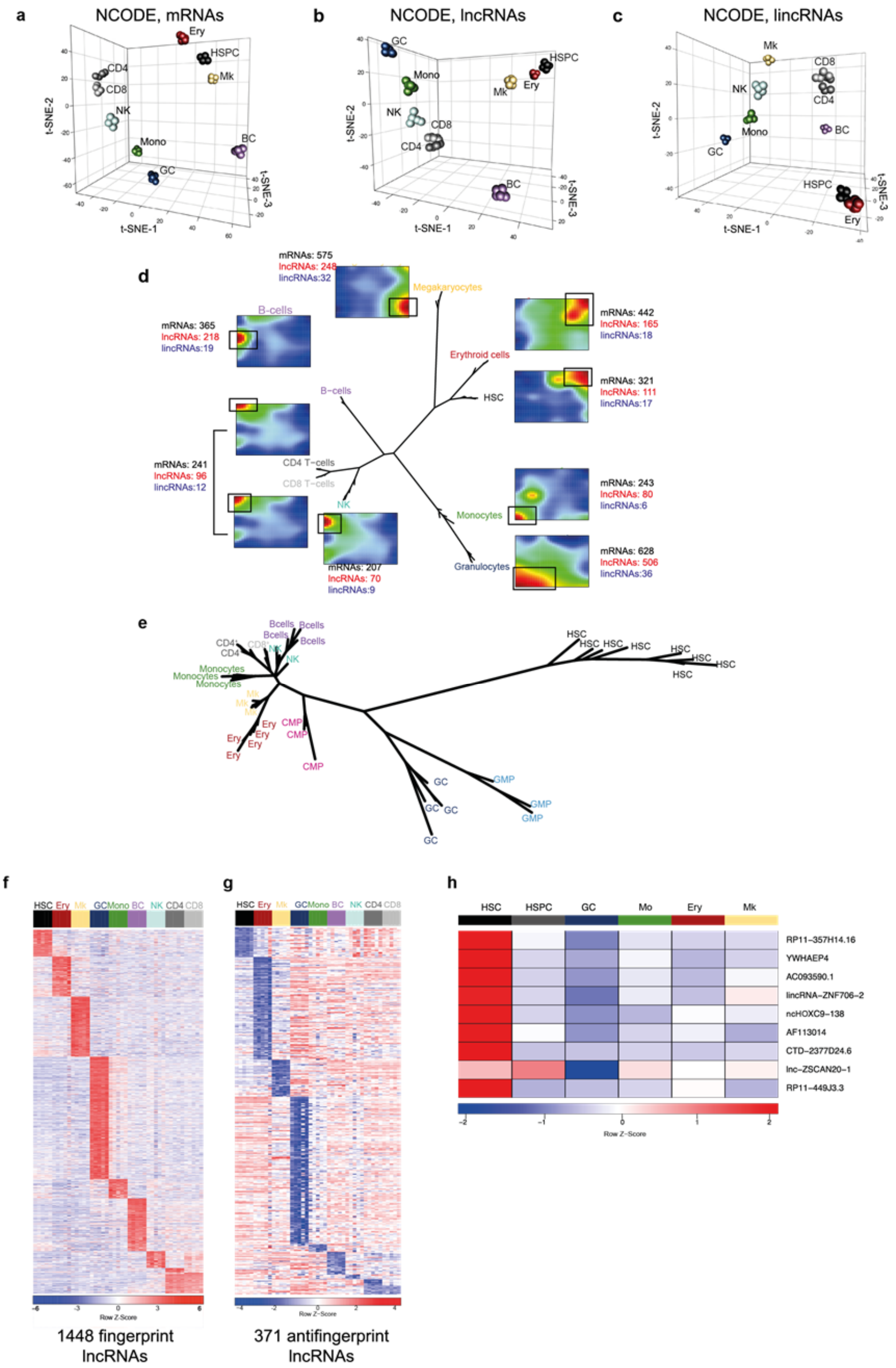

Supplementary Fig. 2. Distinct ncRNA expression profiles characterize cells of the human blood lineages.

All depicted data in (**a-d, f-g**) refer to the NCode™-ncRNA platform. (**a-c**) *t*-SNE of all samples using (**a**) 3,767 ROSE-selected mRNAs (see Materials and Methods for feature selection algorithm), (**b**) 1,512 ROSE-selected ncRNAs and (**c**) 147 ROSE-selected lincRNAs. (**d**) Self organizing maps (SOM) trained using 14,256 ROSE-selected transcripts (mRNAs and ncRNAs) in 9 sample groups as input to the oposSOM package. Black rectangles highlight group-specific overexpression spots. Center: neighbor-joining tree built using 30 lineage-associated spot metagenes identified by oposSOM.[3] (**e**) Neighbor-joining tree based on the expression of 312 spot metagenes identified by oposSOM algorithm from a self-organizing map trained with 5,545 ROSE-selected ncRNAs as input. Heatmaps of (**f**) 1,448 fingerprint ncRNAs and (**g**) 581 anti-fingerprint ncRNAs for each cell type (**h**) Heatmap of qRT-PCR validation results for 9 HSC-specific ncRNAs. Results from three replicate runs were averaged and plotted in the heatmap.

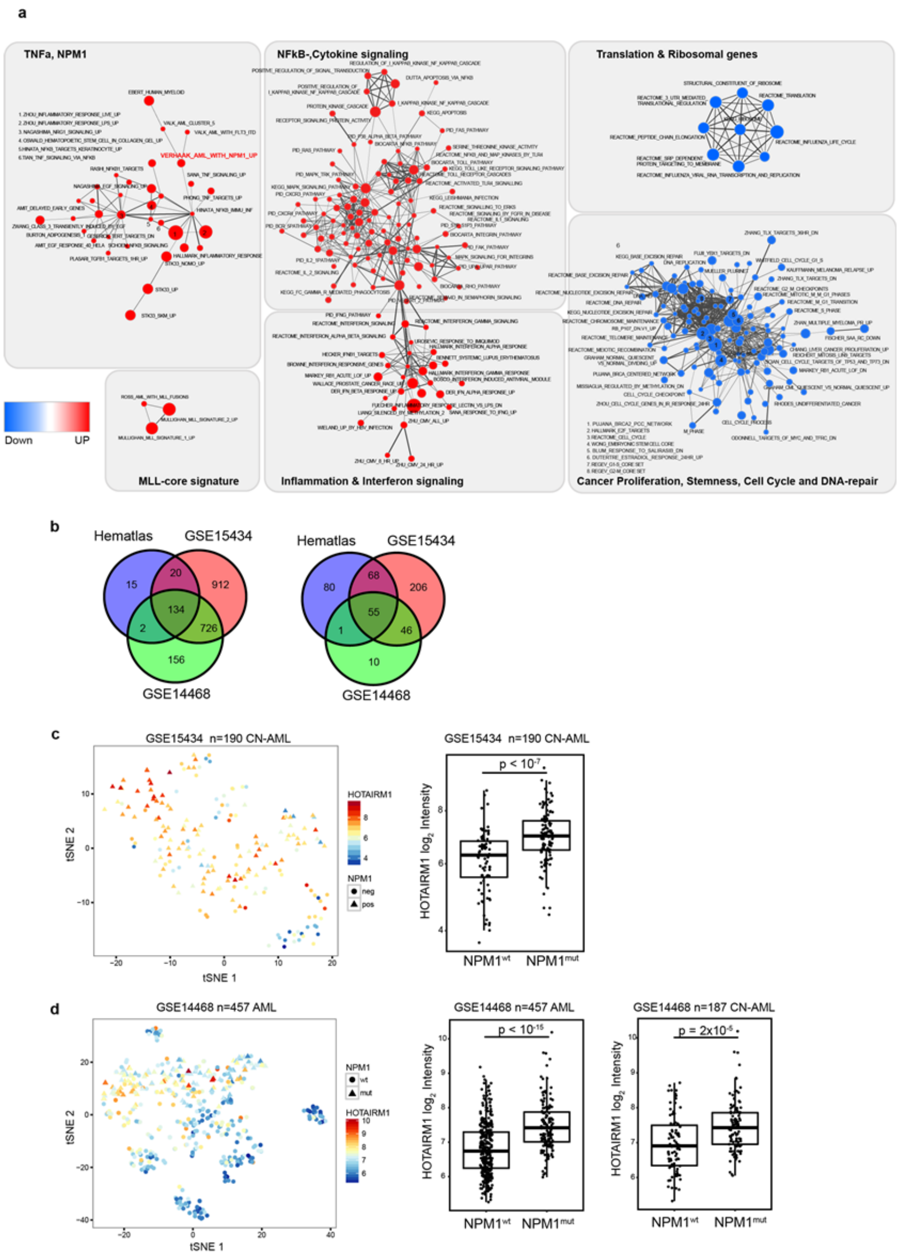

Supplementary Fig. 3. Guilt by association – validation in GSE15434 and GSE14468.

(**a**) Functional clustering of gene sets comprised of coding genes whose expression profiles were correlated or anti-correlated to *HOTAIRM1* in n=190 normal-karyotype (CN-) AML patients in GSE15434 (FDR<0.01). Circle size corresponds to the size of the gene set, and connecting line thickness represents the degree of similarity between two gene sets. (**b**) Overlap in gene sets found to be correlated or anti-correlated to *HOTAIRM1* (FDR<0.05) between our experimental data set (lncScape) and the AML datasets GSE15434 and GSE14468. (**c**) *HOTAIRM1* expression in *NPM1*-mutated AMLs compared to *NPM1*-wild type samples in GSE15434. Left: color-coded *t*-SNE map of n=190 CN-AML; right: group wise *HOTAIRM1* expression. (**d**) *HOTAIRM1* expression in *NPM1*-mutated AMLs compared to *NPM1*-wild type samples in GSE14468 for all patients. Left: color-coded *t*-SNE map of n=457 AML samples; middle: group wise *HOTAIRM1* expression of all n=457 AML patients; right: *HOTAIRM1* expression in n=187 CN-AML samples. P-values were calculated using the two-sided Welch's two sample t test.

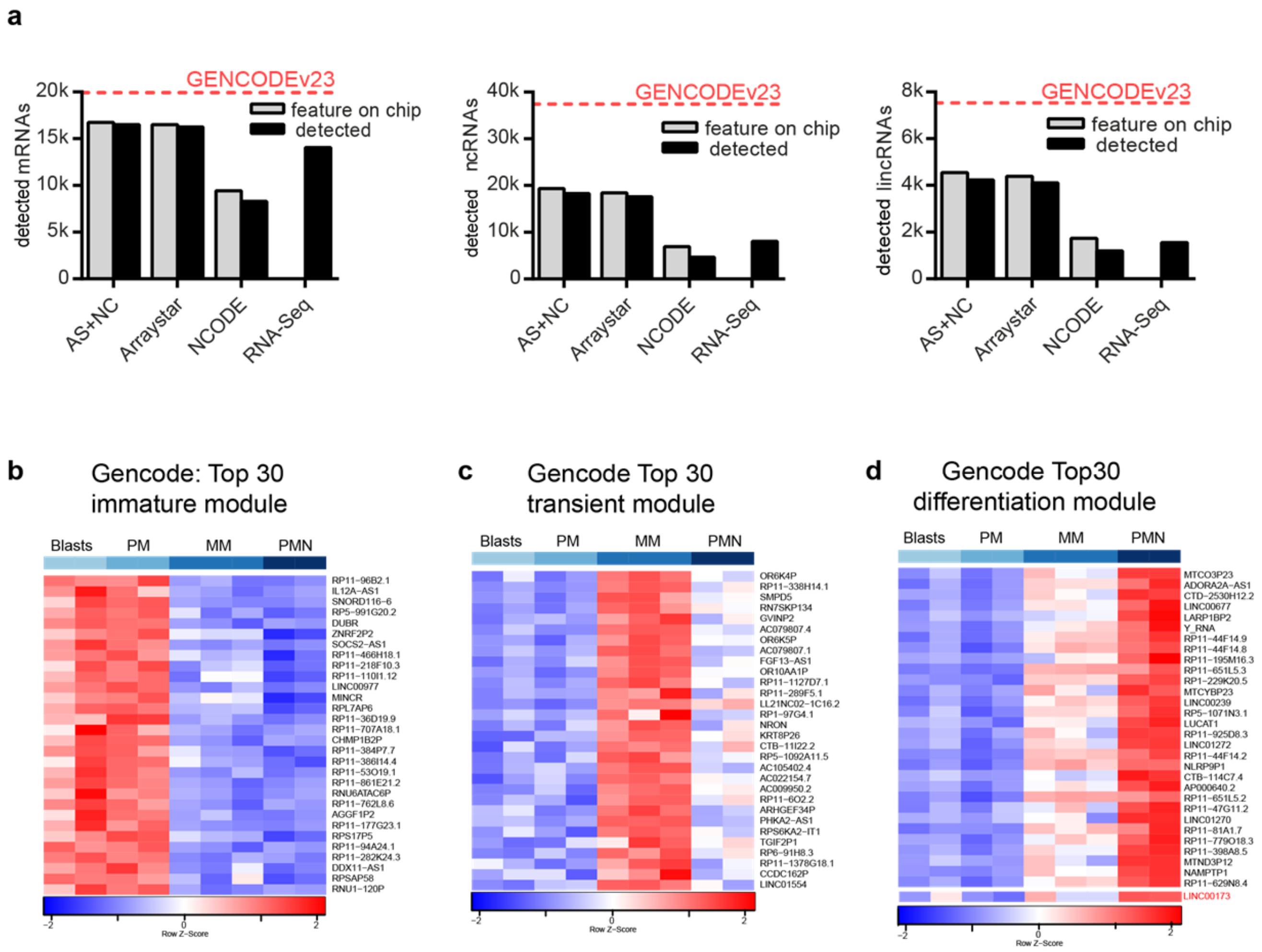

Supplementary Fig. 4. RNASeq-based ncRNA landscape of human myelopoiesis.

(**a**) Number of features that cover and detect GENCODE.v23-annotated mRNAs (left), ncRNAs (middle) and lincRNAs (right) by the Microarray platforms employed in this study (AS+NC: Arraystar and NCODE combined) and RNASeq after filtering for minimal expression (at least 15 total reads) (**b-d**) Heatmap representations of the Top30 GENCODE-annotated ncRNAs in the (**b**) immature module, (**c**) the transiently upregulated module and (**d**) in the differentiation module plus *LINC00173*.

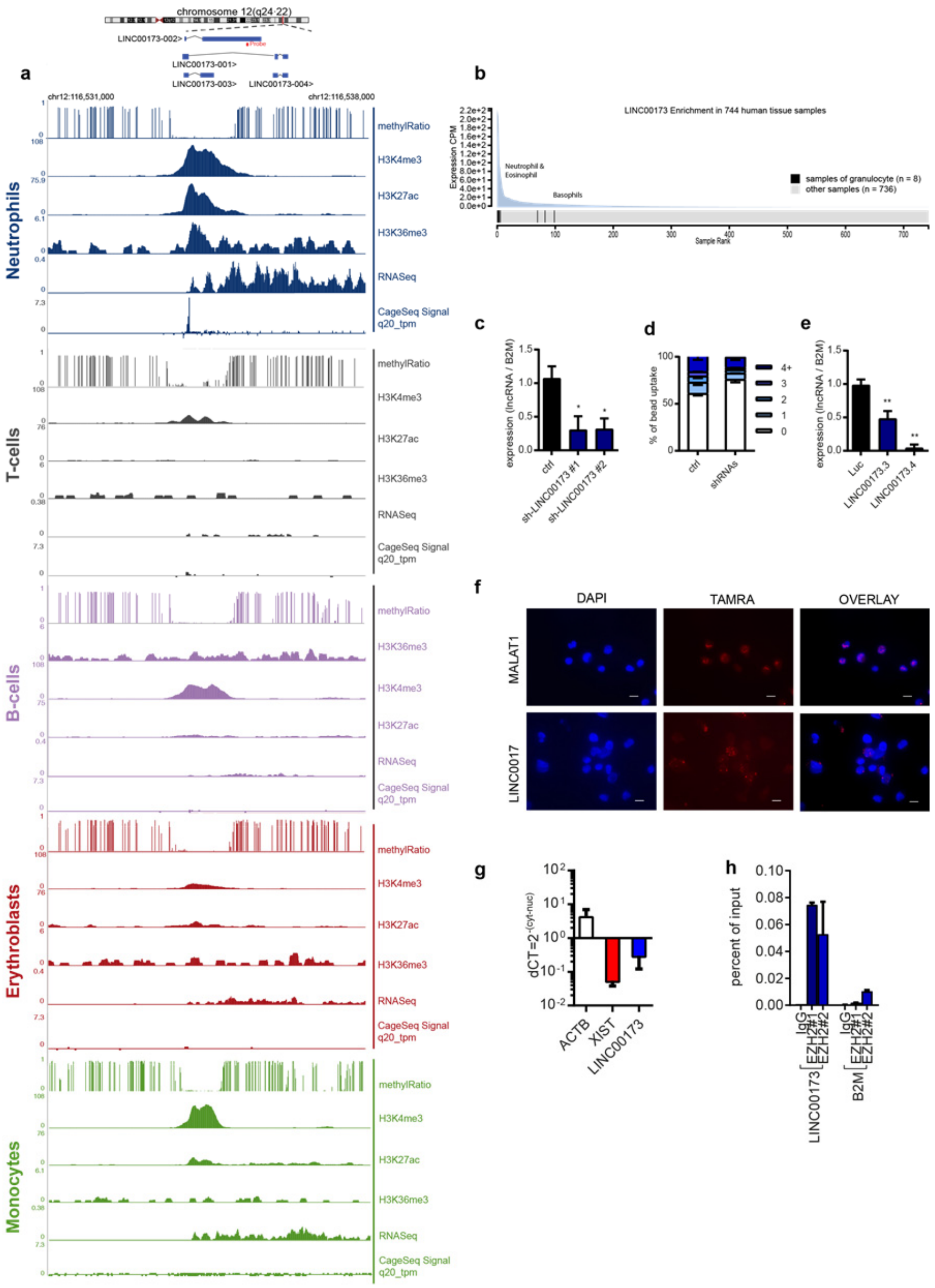

Supplementary Fig. 5. *LINC00173* is a granulocyte-specific lincRNA.

(**a**) The *LINC00173* gene locus depicting the array probe and alternative isoforms (according to ENSEMBL GRCh38.p5) together with UCSC genome browser tracks (GRCh38/hg38) of Methylation profgiles, ChIP-seq/ RNA-seq data (BLUEPRINT)[4], CAGE-seq Signals (FANTOM5)[5] and sequence conservation (GERP-elements)[6] in human blood cells. (**b**) Rank ordered *LINC00173* expression in 744 human tissue samples from the FANTOM5 database[5]. (**c**) Expression of *LINC00173* in shRNA-transduced cells on day 4 of granulocytic *in vitro* differentiation (n=3). (**d**) Phagocytosis assay, quantification of histogram peaks from Fig. 4b. (**e**) Expression of *LINC00173* after CRISPRi in NB4:dCas9-KRAB clones (n=6). (**f**) RNA FISH with tiled biotinylated probes in *in vitro* differentiated granulocytes; scale bars 10µm. (**g**) QRT-PCR analysis of fractionated RNA of *in vitro* differentiated granulocytes. The cytoplasmic:nuclear ratio is shown. (**h**) RIP in THP-1 cells using two different antibodies, followed by qRT-PCR to detect binding if EZH2 to *LINC00173*. Data are presented as percent of input in comparison to *B2M*. (**c-e, g-h**) Data are presented as mean ± s.d. *P<0.05; **P<0.01; ns, not significant; unpaired t test with Holm-Sidak correction.

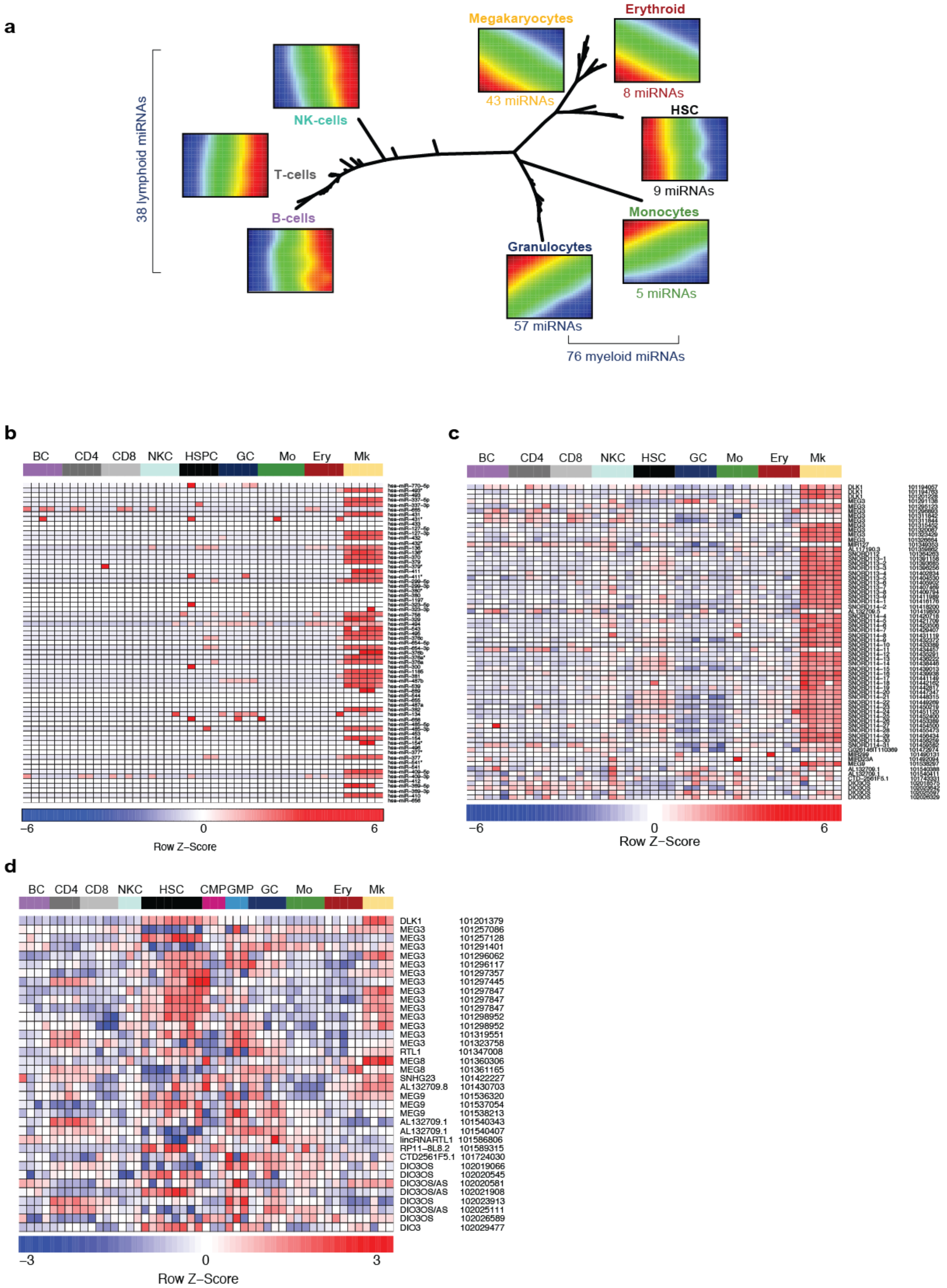

Supplementary Fig. 6. (**a**) Neighbor-joining tree based on the expression of spot metagenes identified by oposSOM algorithm from a self organizing map trained with all 242 expressed miRNAs as input. (**b-d**) NcRNAs of the *DLK1-DIO3* locus are upregulated in megakaryocytes. (**b**) Heatmap of NCode™-miRNA microarray probe sets covering the *DLK1-DIO3* locus in the order of ascending genomic location, showing megakaryocyte-specific expression for most members of the miR-127~136 and miR-379~410 clusters. (**c**) Heatmap of all probe sets covering the *DLK1-DIO3* locus on the NCode™-ncRNA Array in the order of ascending genomic location showing megakaryocyte-specific expression of *DLK1*, *MEG3*, *MEG8*, *MEG9* and the embedded snoRNA cluster. (**d**) Heatmap of Arraystar lncRNA V2.0 probe sets covering the *DLK1-DIO3* locus showing high expression of the region between *DLK1* and *MEG9* in HSCs and megakaryocytes.

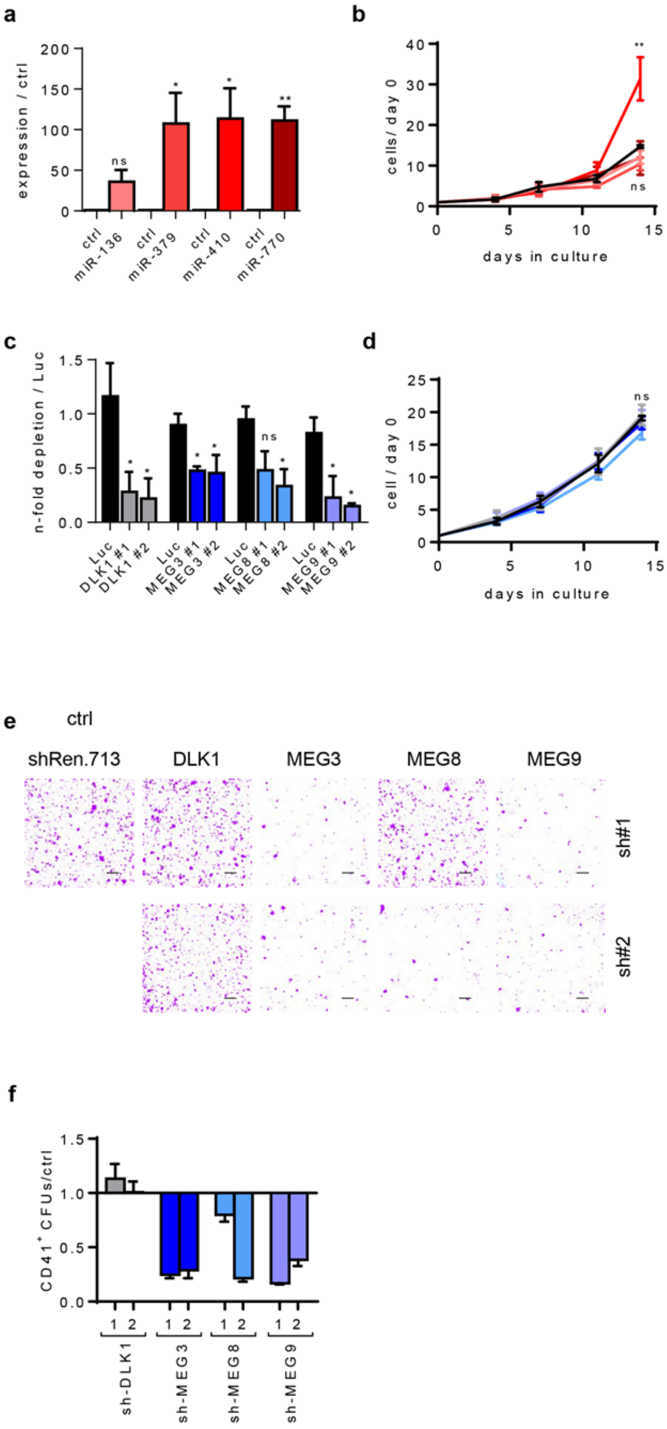

Supplementary Fig. 7. NcRNAs of the DLK1-DIO3-locus control human megakaryopoiesis. (**a**) QRT-PCR of miRNA overexpression in CD34$^+$ cells during megakaryocytic/ erythroid differentiation (n=4). (**b**) Number of CD34$^+$ miRNA-transduced cells during megakaryocytic/erythroid *in vitro* differentiation (normalized to day 0). (**c**) QRT-PCR validation of shRNA-mediated *DLK1*, *MEG3*, *MEG8* and *MEG9* knockdown in CD34$^+$ cells during megakaryocytic/ erythroid differentiation (n=5). (**d**) Number of shRNA-transduced cells during megakaryocytic/erythroid *in vitro* differentiation. The cell counts were normalized to day 0; data are shown as the mean of two replicates per shRNA. (n=5). (**e**) Images of collagen-based CFU-Mk assays (whole slides) stained for CD41$^+$ CFU-Mks on day 14; scale bars 2mm. (**f**) Automated scanning-microscopy-based quantitation of CD41$^+$ colonies in collagen-based CFU-Mk assays (as shown in **e**) (n=3). (**a-d, f**) Data are presented as mean ± s.e.m. *P<0.05; **P<0.01; ns, not significant; One-way ANOVA with Dunnett's post hoc test.

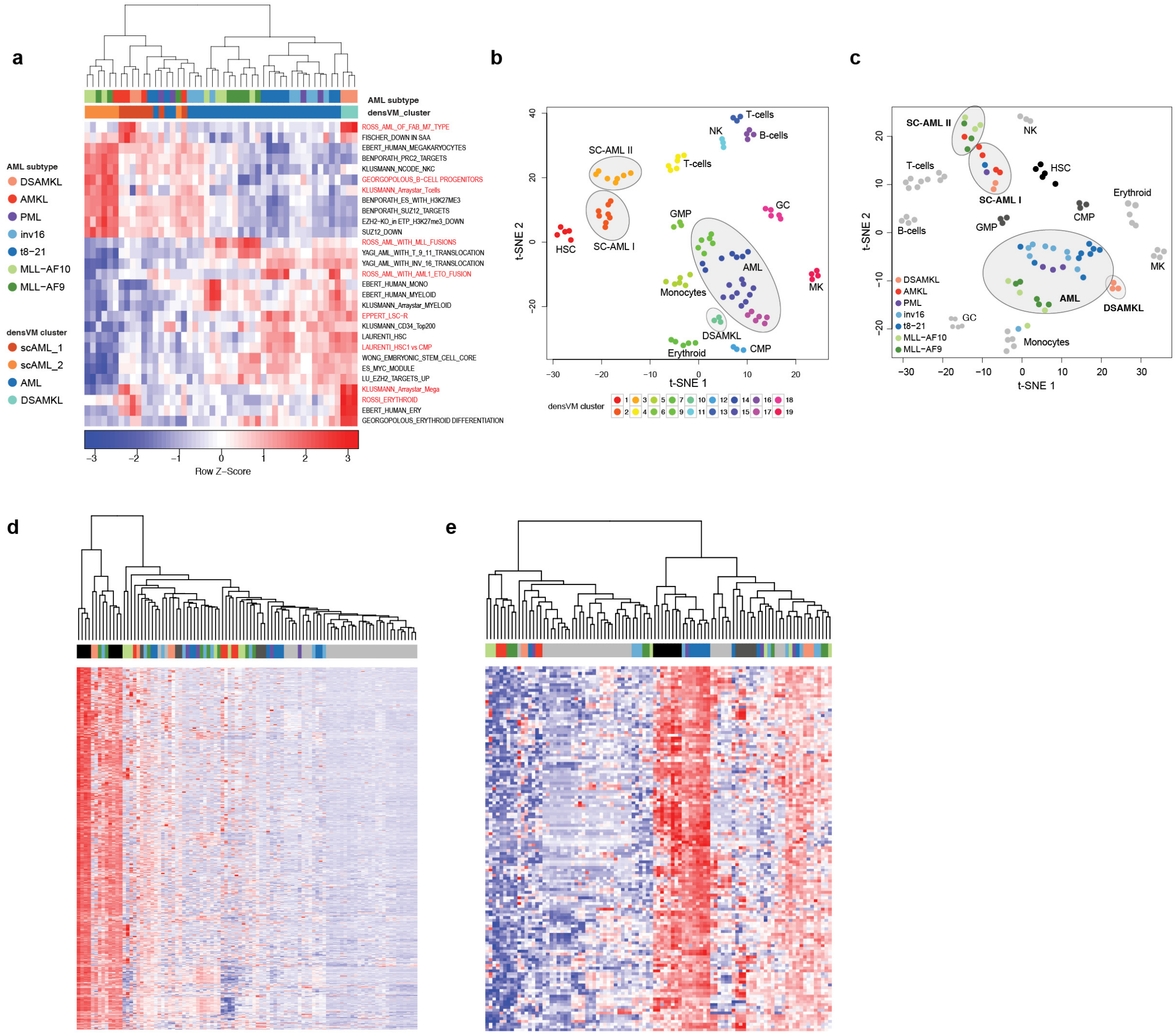

Supplementary Fig. 8. Integrated analysis reveals ncRNA stem cell signature in AML and HSCs. (**a**) Unsupervised clustering of pathway activity scores[7] performed on the AML samples, showing the pathway activity of the indicated gene sets in all AML samples. The analysis demonstrates the enrichment of lymphoid and erythroid gene sets in the SC-AML I/II clusters, the upregulation of *MLL*-fusion associated gene sets in the *MLL*-rearranged AML samples not belonging to the SC-AML clusters, and the enrichment of AML1-ETO target genes in the t(8;21) samples. (**b**) densVM-clustering[8] structures the *t*-SNE representation in Fig. 6b into 11 healthy populations and 4 AML clusters. The samples in the cluster "AML" were comprised of 2-5 subclusters, depending on number of ncRNA features used in the *t*-SNE analysis. In all runs these clusters were immediate neighbors and were therefore merged into one bigger cluster. (**c**) *t*-SNE analysis using the 5,255 most variable ncRNAs as input, showing the stability of the clustering independent of the number of ncRNAs used as input. (**d**) Unsupervised clustering of 1215 ncRNAs assigned to Spot A, showing strong expression in HSCs and in AMKL and *MLL*-rearranged samples and absence from differentiated cells (grey). (**e**) Unsupervised clustering of 300 ncRNAs assigned to Spot B showing strong expression in HSCs and most notably in t(8;21) AML samples, as well as in some AMKL and MLL samples.

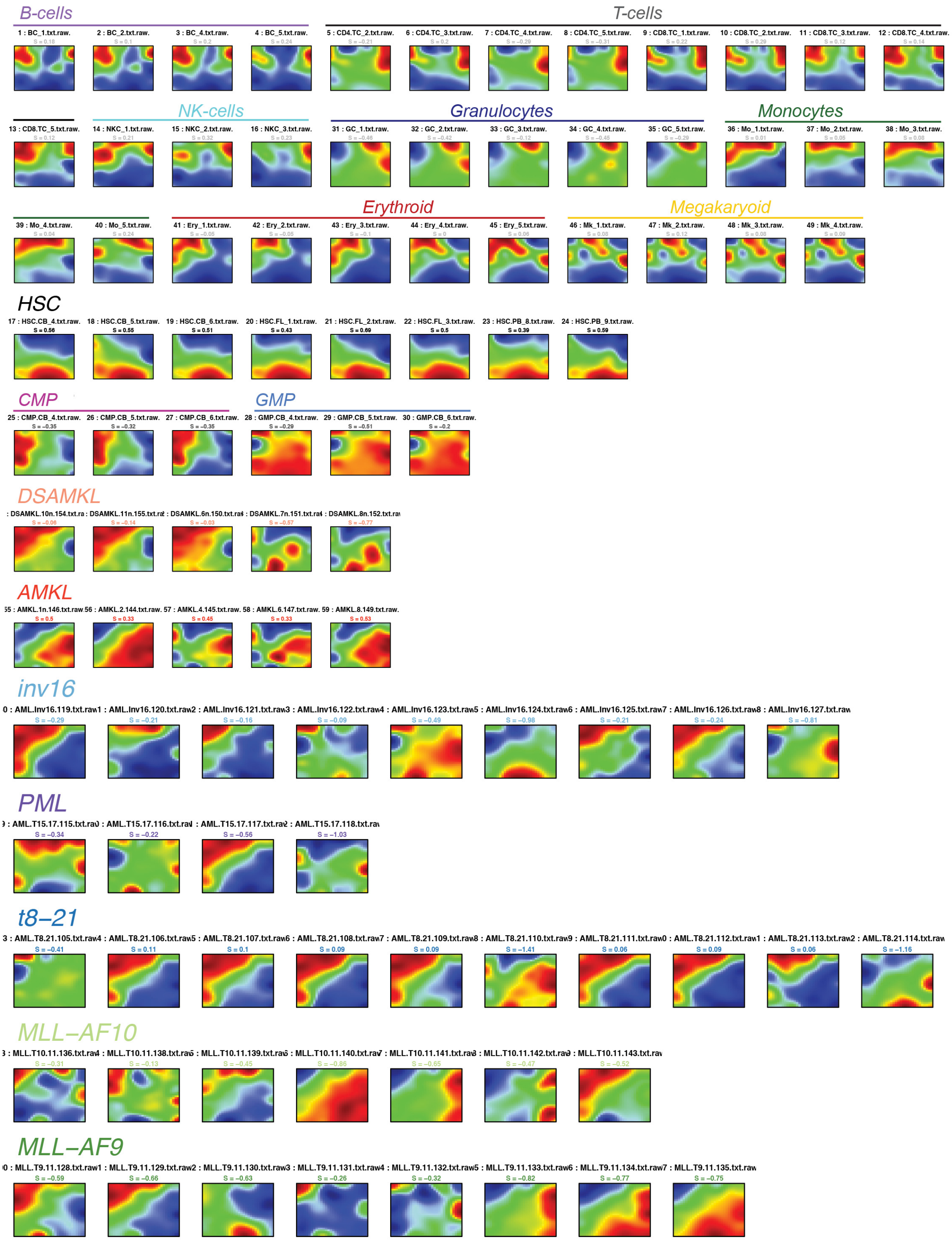

Supplementary Fig. 9 SOM expression profiles of all samples in the landscape resource. Expression portraits of the AML samples were obtained by training a SOM with the 7094 most variable ncRNAs as input into the oposSOM analysis[3] pipeline.

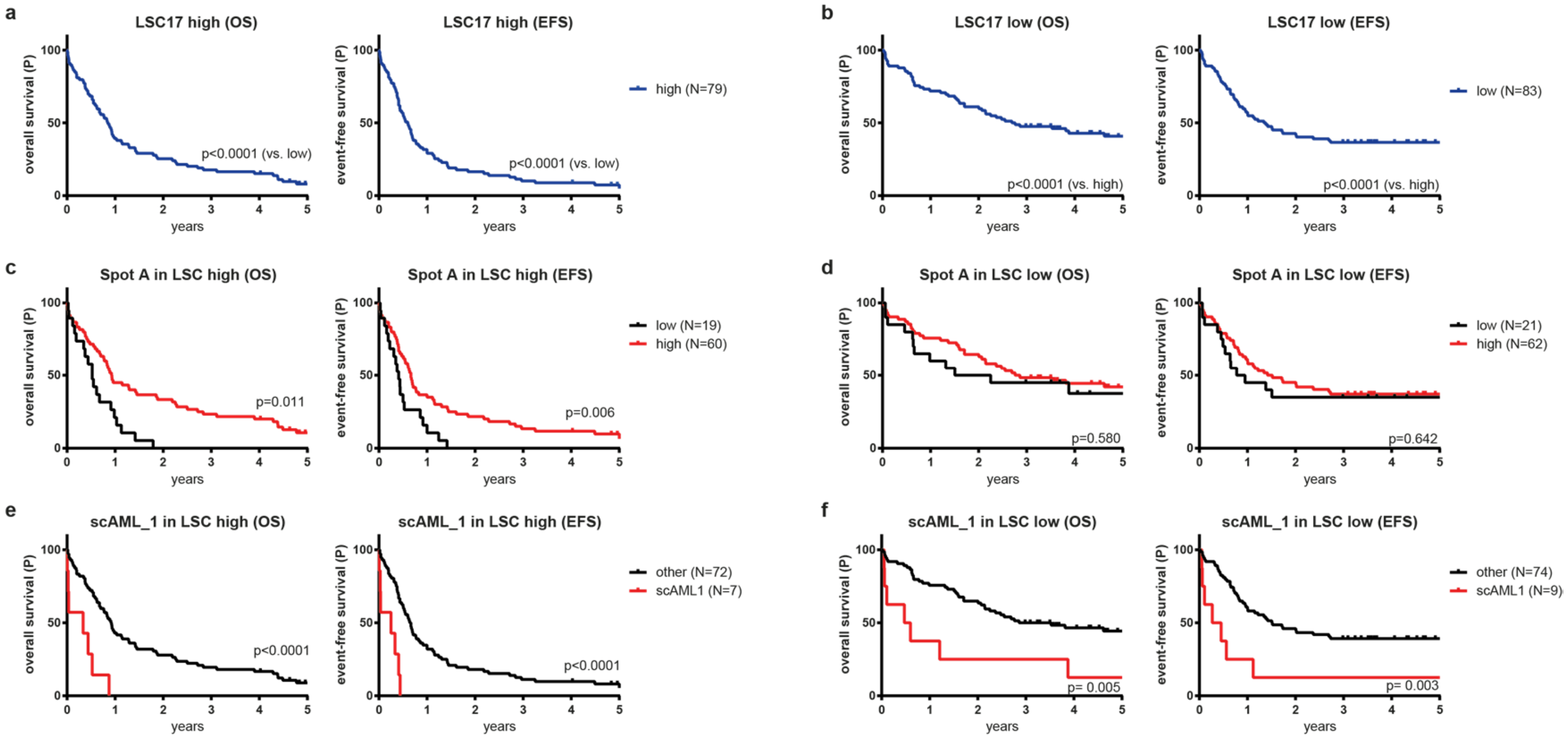

Supplementary Fig. 10 (**a-f**) 5-year overall survival (left) and event-free survival (right) of 162 adult AML samples[9] with a high (a, c and e) or low (b, d and f) calculated LSC17 score[10] (a, c, e: "LSC high"; b, d, f: "LSC low") grouped according to their of Spot A (**c-d**) or SC-AML ncRNA expression profile (**e-f**) using unsupervised k-means clustering.

Supplementary Table 1: Patient characteristics of the TCGA cohort

**Sex - no. (%)**	
Male	54.00
Female	46.00
N/A	0.00
**Age - year**	
Median	57.00
Range	18 - 88
>= 60 (%)	45.55
**Overall Survival - year**	
Median	1.39
Range	0-10.708
**Whitelcell count - x10^9/L**	
Median	16.15
Range	0.4-298.4
**FAB type - no. (%)**	
M0	9.50
M1	23.00
M2	22.00
M3	10.00
M4	20.50
M5	11.00
M6	1.50
M7	1.50
Not determined/Other	1.00
**Cytogenetic abnormalities - no. (%)**	
t(15;17)	9.00
t(8;21)	3.50
inv(16)/t(16;16)	5.50
t(9;11)	1.00
t(11q23) (Note: not including t(9;11))	0.00
-7/del(7q)	6.50
-5/del(5q)	8.00
-3/inv(3)/t(3;3)	3.50
t(6;9)	0.00
Complex karyotype	12.00
Other abnormal karyotype	19.00
Cytogenetic normal	42.50
Not determined	1.50
**Molecular abnormalities − no. (%)**	
FLT3-ITD or -TKD	28.00
N- or K-RAS	13.00
NPM1	27.00
CEBPA	6.50
**ELN - no. (%)**	
Favorable	28.50
Intermediate-I	25.00
Intermediate-2	12.50
Adverse	22.00

Supplementary References

1. Lee, J. K. *et al*. Comparing cDNA and oligonucleotide array data: concordance of gene expression across platforms for the NCI-60 cancer cells. *Genome Biol.* **4**, R82 (2003).

2. Novershtern, N. *et al*. Densely interconnected transcriptional circuits control cell states in human hematopoiesis. *Cell* **144**, 296-309 (2011).

3. Loffler-Wirth, H., Kalcher, M. & Binder, H. oposSOM: R-package for high-dimensional portraying of genome-wide expression landscapes on bioconductor. *Bioinformatics* **31**, 3225-3227 (2015).

4. Martens, J. H. & Stunnenberg, H. G. BLUEPRINT: mapping human blood cell epigenomes. *Haematologica* **98**, 1487-1489 (2013).

5. Hon, C. C. *et al*. An atlas of human long non-coding RNAs with accurate 5' ends. *Nature* **543**, 199-204 (2017).

6. Davydov, E. V. *et al*. Identifying a high fraction of the human genome to be under selective constraint using GERP++. *PLoS Comput. Biol.* **6**, e1001025 (2010).

7. Lee, E., Chuang, H. Y., Kim, J. W., Ideker, T. & Lee, D. Inferring pathway activity toward precise disease classification. *PLoS Comput. Biol.* **4**, e1000217 (2008).

8. Becher, B. *et al*. High-dimensional analysis of the murine myeloid cell system. *Nat. Immunol.* **15**, 1181-1189 (2014).

9. Cancer Genome Atlas Research Network. Genomic and epigenomic landscapes of adult de novo acute myeloid leukemia. *N. Engl. J. Med.* **368**, 2059-2074 (2013).

10. Ng, S. W. *et al*. A 17-gene stemness score for rapid determination of risk in acute leukaemia. *Nature* **540**, 433-437 (2016).
